# Antimicrobial resistance and genotyping of *Pseudomonas aeruginosa* isolated from the ear canals of dogs in Japan

**DOI:** 10.3389/fvets.2023.1074127

**Published:** 2023-07-20

**Authors:** Ahmed Elfadadny, Jumpei Uchiyama, Kazuyoshi Goto, Ichiro Imanishi, Rokaia F. Ragab, Wedad M. Nageeb, Keita Iyori, Yoichi Toyoda, Toshihiro Tsukui, Kaori Ide, Keiko Kawamoto, Koji Nishifuji

**Affiliations:** ^1^Laboratory of Internal Medicine, Cooperative Division of Veterinary Sciences, Graduate School of Agriculture, Tokyo University of Agriculture and Technology, Fuchu, Japan; ^2^Department of Animal Internal Medicine, Faculty of Veterinary Medicine, Damanhour University, Damanhour, Egypt; ^3^Department of Bacteriology, Graduate School of Medicine Dentistry and Pharmaceutical Sciences, Okayama University, Okayama, Japan; ^4^Department of Microbiology, Kitasato University School of Medicine, Sagamihara, Japan; ^5^Department of Medical Microbiology and Immunology, Faculty of Medicine, Suez Canal University, Ismailia, Egypt; ^6^Dermatological and Laboratory Service for Animals, Vet Derm Tokyo, Fujisawa, Japan; ^7^Zenoaq Co. Ltd., Koriyama, Japan; ^8^Division of Animal Life Science, Institute of Agriculture, Graduate School, Tokyo University of Agriculture and Technology, Fuchu, Japan; ^9^Laboratory of Immunology and Infection Control, Department of Veterinary Medicine, School of Veterinary Medicine, Azabu University, Sagamihara, Japan

**Keywords:** otitis, dog, antimicrobial resistance, multilocus sequence typing, *Pseudomonas aeruginosa*

## Abstract

The strong bond between dogs and their owners creates a close association that could result in the transfer of antibiotic-resistant bacteria from canines to humans, potentially leading to the spread of antimicrobial resistance genes. *Pseudomonas aeruginosa*, a common causative agent of persistent ear infections in dogs, is often resistant to multiple antibiotics. Assessing the antimicrobial resistance profile and genotype of *P. aeruginosa* is crucial for the appropriate use of veterinary pharmaceuticals. However, in recent years, few studies have been conducted on this bacterium in Japan. We determined the antimicrobial resistance profile and genotype of *P. aeruginosa* isolated from the ear canal of dogs in Japan in 2020. Analysis of antimicrobial resistance using disk diffusion tests indicated a high frequency of resistance to most antimicrobial agents. Particularly, 29 isolates from the ear canals of the 29 affected dogs (100%) were resistant to cefovecin, cefpodoxime, and florfenicol; however, they were susceptible to cefepime and piperacillin/tazobactam. Only 3.4, 10.3, and 10.3% of the isolates were resistant to ceftazidime, tobramycin, and gentamicin, respectively. Furthermore, upon analyzing the population structure using multilocus sequence typing, a considerably large clonal complex was not observed in the tested isolates. Three isolates, namely ST3881, ST1646, and ST532, were clonally related to the clinically isolated sequence types in Japan (such as ST1831, ST1413, ST1812, and ST1849), which is indicative of dog-to-human transmission. Considering the variation in antibiotic resistance compared to that reported by previous studies and the potential risk of dog-to-human transmission, we believe that the survey for antimicrobial resistance profile and population structure should be continued regularly. However, the prevalence of multidrug-resistant *P. aeruginosa* in dogs in Japan is not a crisis.

## Introduction

Canine otitis is one of the most commonly reported issues under the dermatology section of veterinary hospitals (1). It encompasses two common types: otitis externa, which refers to inflammation of the outer ear canal and pinna, and otitis media, which involves inflammation of the middle ear (2, 3). In dogs, otitis externa occurs more commonly than otitis media. However, in certain cases, otitis externa can progress and penetrate the tympanic membrane, thereby spreading the infection to the middle ear and leading to otitis media (2, 4). The major causes of canine otitis are the prolonged exposure of the canine to an etiological agent within its environment and/or the failure of the antimicrobial treatment in eliciting a response from the microbes, when the disease is complicated with bacterial strains (4). Repeated episodes of inflammation can cause severe histopathological changes in the ear canal. These changes include epithelial and glandular hyperplasia, calcification, and increased cerumen production along the external ear canal, which can lead to further failure of otitis treatment and potentially result in end-stage ear infection (5).

Canine otitis also has a detrimental effect on the owner’s quality of life (6). The prevalence of otitis cases that are diagnosed in clinical veterinary practice ranges from 8.7 to 20% for otitis externa (7–9) and 50 to 80% for otitis media, as a complication to external otitis (10). In a one-year-prevalence study conducted in the United Kingdom, the prevalence of otitis was notably higher in certain canine species, such as Basset Hound (28.81%), Chinese Shar Pie (17.76%), Labradoodle (17.71%), Beagles (14.72%) Golden Retrievers (14.11%), and Cockapoo (12.97%) (11). In Japan, the incidence of otitis in dogs admitted to the animal hospital in Osaka prefecture was primarily confined to miniature poodles and cocker spaniel dogs (12). The incidence of otitis in a specific species has been attributed to the anatomical confirmation of the ear pinnae and ear canal (13). In a previous study (11), the incidence odds of otitis were 1.76 and 1.84 times in the breeds with pendulous and V-shaped drop pinnae, respectively, compared to that in breeds with the erect-shaped ear. These results suggested that the retention of heat and moisture, as well as accumulation of foreign materials in pendulous and V-shaped drop pinnae may provide a favorable environment for bacterial colonization (14, 15). Canine otitis is driven by secondary bacterial pathogens in 98% of the cases in North America (16, 17), especially coagulase-positive *Staphylococcus* species, *Pseudomonas*, *Proteus*, *Escherichia coli*, *Klebsiella*, and *Malassezia* species, and mixed infections are common (10, 18). The major organisms isolated from dogs suffering from otitis are *Staphylococcus* spp., especially *S. pseudintermedius*, which account for 10–70% of cases (19). *Pseudomonas aeruginosa* is the second major otitis pathogen, which accounts for 20–60% of cases and can cause intractable otitis (4, 19–21). The canine otitis caused by *P. aeruginosa* is challenging to cure because of its biofilm formation and intrinsic and acquired drug resistance (22).

Previous studies have demonstrated that the extensive utilization of antimicrobial agents in pets, especially in dogs and cats, may pose a potential threat to public health because of the likelihood of such pets serving as a means for the spread of antimicrobial-resistant bacteria and elements (23). A study demonstrated that the resistance of *P. aeruginosa* to antimicrobials includes not only penicillin, aminoglycosides, and fluoroquinolones groups (24), but also recently formulated drugs, such as ceftolozone-tazobactam and ceftazidime-avibactam (25, 26). Such antimicrobial resistance leads to challenges in the treatment of otitis caused by *P. aeruginosa* in dogs in the clinical setting (27). Thus, studies are required to monitor the efficacy of drugs used in dogs infected by *P. aeruginosa* to maintain the potential of a critical therapeutic agent and develop an effective management plan for the long run (28, 29). Several molecular typing techniques have been used to type *P. aeruginosa* strains, such as pulsed-field gel electrophoresis (30), polymerase chain reaction (PCR) fingerprinting (31), and multilocus sequence typing (MLST) (32). Among these techniques, MLST was developed in 2004 for *P. aeruginosa*, and this method is based on the sequencing of the allelic difference of seven housekeeping genes, which collectively provide the sequence types (STs) to characterize isolates. The selection of the seven loci allows scientists to track variability among strains such that one can be different in one or more loci and still be included in the global clonal history of the species with the highest possible accuracy (32, 33).

The One Health Perspective approach links the safety and health of humans with that of their surroundings (34), and this approach has recently raised the consideration of a survey for antimicrobial drug–resistant bacteria in small animals (35). The close contact between pets and humans offers excellent opportunities for the interspecies transmission of resistant bacteria in either direction, such as through licking, petting, and physical injuries. In contrast to other diseases, the horizontal transmission of antimicrobial resistance genes could occur either in the human host and/or environment. For example, *P. aeruginosa* bacterium of dogs and humans can meet outside their hosts and allow the transfer of resistance genes (23). In comparison to food-borne zoonoses, pet-associated zoonoses remain a neglected area globally. A few epidemiological studies on the antimicrobial resistance profile of *P. aeruginosa* in companion animals have been conducted in Japan (36, 37). Despite the importance of conducting a continuous survey on this topic, which should involve a large number of antibiotics, there has been a scarcity of studies in recent years. Our study investigated the antimicrobial resistance profile and genotype structure of *P. aeruginosa* isolated from ear canals of dogs in Japan during the year 2020, to determine their genetic relationship to other *P. aeruginosa* clones circulating in Japan.

## Materials and methods

### Animals and bacterial isolation

The samples were collected using sterile swabs from the ear canals of 29 dogs affected by unilateral and/or bilateral otitis, which were diagnosed from January to December 2020 by local veterinarians in Japan. The swabs were transported to VDT Co. Ltd. after being placed in the Stuart transport medium (Copan Diagnostics Inc., United States). Furthermore, the swabs were immediately smeared onto cetrimide agar (Wako, Fujifilm, Japan), a selective media for *P. aeruginosa* species identification, and incubated overnight at 35–37°C. The isolated bacteria were identified using a Microflex system (Bruker Daltonics, Bremen, Germany). The protein profile of each bacterium was analyzed using the matrix-assisted laser desorption ionization (MALDI) Biotyper v3.1.

The signalment information of dogs, including breed, age, and sex is listed in Table 1. The Biosafety Management Committee of the Tokyo University of Agriculture and Technology reviewed and approved all experiments using *P. aeruginosa* strains (#R2-10).

**Table 1:** Signalment information of 29 *Pseudomonas aeruginosa* isolates obtained from the ear canals of dogs in Japan.

Strain ID	Date of sampling^a^	Breed	Sex^b^	Age (year)	Previous antibiotic use^c^	Resistance profile	Susceptibility pattern^d^	STs
PA 22861	01/07/2020	Cavalier King Charles Spaniel	CM	7	GM	CPDX, CFV, AZT, FRM, AMK, LVLX, FF	XDR	3881
PA 23303	01/21/2020	Border Collie	CM	6	GM, FF	CPDX, CFV, AZT, AMK, CIP, LVLX, FF	XDR	3882
PA 23405	01/25/2020	Yorkshire terrier	CM	7	ERFX, DOXY	CAZ, CPDX, CFV, AZT, IPM, GM, FRM, AMK, CIP, OFLX, LFLX, LVLX, OBFX, ERFX, FF	XDR	3883
PA 23824	02/10/2020	Toy poodle	M	7	CEX, FF	CPDX, CFV, AMK, CIP, FF	XDR	643
PA 23943	02/13/2020	Petit Basset Griffon Vendeen	CM	6	none	CPDX, CFV, CIP, FF	MDR	654
PA 24223	02/21/2020	Golden retriever	CM	4	none	CPDX, CFV, AZT, AMK, CIP, LVLX, FF	XDR	3884
PA 24374	02/26/2020	Labrador Retriever	SF	8	none	CPDX, CFV, CIP, FF	MDR	2291
PA 24608	03/04/2020	Basset hound	CM	8	none	CPDX, CFV, CIP, FF	MDR	645
PA 24667	03/06/2020	Frenchi Bulldog	SF	7	FOM	CPDX, CFV, AZT, AMK, CIP, OFLX, LFLX, LVLX, OBFX, ERFX, FF	XDR	1153
PA 25065	03/18/2020	Japanese spaniel	SF	12	AMPC, MINO	CPDX, CFV, FF	non-MDR	1741
PA 25268	03/21/2020	Standard poodle	SF	9	none	CPDX, CFV, AZT, AMK, LVLX, FF	XDR	1790
PA 27532	05/17/2020	Clumber spaniel	M	12	MEPM, FRPM	CPDX, CFV, AZT, AMK, CIP, OFLX, LFLX, LVLX, OBFX, ERFX, FF	XDR	1646
PA 27567	06/07/2020	Boston terrier	CM	13	none	CPDX, CFV, CIP, LVLX, FF	MDR	3885
PA 27976	06/08/2020	Giant Schnauzer	F	5	OBFX	CPDX, CFV, FF	non-MDR	2839
PA 30241	06/19/2020	Chihuahua	SF	8	ERFX	CPDX, CFV, AMK, CIP, LVLX, FF	XDR	377
PA 30323	08/16/2020	Cavalier King Charles Spaniel	SF	12	none	CPDX, CFV, AZT, FRM, AMK, CIP, OFLX, LFLX, LVLX, OBFX, ERF, FF	XDR	1153
PA 30580	08/12/2020	Shiba inu	M	12	CIP	CPDX, CFV, FRM, CIP, LVLX, LFLX, OBFX, FF	XDR	313
PA 32450	08/18/2020	Shiba inu	M	11	none	CPDX, CFV, AZT, CIP, LVLX, OBFX, FF	MDR	3005
PA 33146	10/03/2020	Miniature Schnauzer	M	12	OBFX	CPDX, CFV, AZT, AMK, CIP, LVLX, OBFX, FF	XDR	2473
PA 33343	10/19/2020	Miniature dachshund	F	5	AMPC/CVA	CPDX, CFV, AZT, AMK, CIP, LVLX, OBFX, FF	XDR	2884
PA 33427	10/22/2020	Maltese	CM	11	CEX	CPDX, CFV, CIP, LFLX, OBFX, ERFX, FF	MDR	266
PA 33543	10/23/2020	Yorkshire terrier	F	6	OFLX, ABPC	CPDX, CFV, CIP, OBFX, ERFX, FF	MDR	560
PA 33568	10/28/2020	Mixbreed	CM	17	CEF	CPDX, CFV, CIP OBFX, ERFX, FF	MDR	505
PA 33599	10/27/2020	Bernese Mountain Dog	F	4	LVFX	CPDX, CFV, AZT, AMK, CIP, ERFX, FF	XDR	2839
PA 33670	10/29/2020	Beagle	CM	6	OBFX, CEX	CPDX, CFV, AZT, TOB, AMK, CIP, OFLX, LFLX, LVLX, OBFX, ERFX, FF	XDR	27
PA 34383	10/31/2020	German Shepherd Dog	M	2	none	CPDX, CFV, AZT, MEM, GM, TOB, FRM, AMK, CIP, OFLX, LFLX, LVLX, OBFX, ERFX, FF	XDR	3886
PA 34599	11/18/2020	Golden retriever	CM	6	none	CPDX, CFV, AZT, TOB, AMK, CIP, LVLX, FF	XDR	532
PA 35113	11/23/2020	Toy poodle	CM	3	none	CPDX, CFV, AZT, CIP, LVLX, OBFX, ERFX, FF	MDR	3887
PA 35671	12/05/2020	Standard poodle	CM	13	FF, CEX	CPDX, CFV, CIP, OFLX, LFLX, LVLX, OBFX, ERFX, FF	MDR	3888

^a^mm/dd/yyyy.

^b^CM, Castrated male; M, Male; F, Female; SF; spayed female.

^c^GM, Gentamicin; FF, Florfenicol; ERFX, Enrofloxacin, DOXY, Doxycycline; CEX, Cefalexin, FOM, Formycin; MINO, Minocycline; OBFX, Orbifloxacin; CIP, Ciprofloxacin; AMPC/CVA, Ampicillin/clavulanic acid; OFLX, Ofloxacin; ABPC; Amoxicillin; CEF, Cefovecin; LVFX, Levofloxacin; COL, colistin; CAZ, ceftazidime; CPDX, cefpodoxime; CFV, cefovecin; AZT, aztreonam; PIP/TAZ, piperacillin-tazobactam; IPM, imipenem; MEM, meropenem; TOB, tobramycin, FRM, fardiomycin; AMK, amikacin; OFLX, ofloxacin; LFLX, lomefloxacin.

^d^MDR, multidrug-resistant; XDR, extensively drug-resistant.

### Antimicrobial susceptibility test

The antibiotic susceptibility test was performed using the disk diffusion and minimum inhibitory concentration (MIC) methods, based on the Clinical and Laboratory Standards Institute guidelines (CLSI) for Vet-08 and M-100-30^th^ED (38). Briefly, the test was carried out using BD sensei-Disk of the designated antibiotic drug (agar diffusion test; Kirby–Bauer disk diffusion method) on bacteria-inoculated Müller–Hinton Agar (Becton Dickinson GmbH, Heidelberg, Germany). We analyzed the antimicrobial resistance patterns of *P. aeruginosa* isolates using a panel of 20 antibiotics, which could be categorized into six types based on their mode of action, including fluoroquinolones, aminoglycoside, cephalosporins, carbapenems, piperacillin-tazobactam, and polymyxin. The following antibiotics were used: colistin (COL), ceftazidime (CAZ), cefpodoxime (CPDX), cefovecin (CFV), cefepime (CFPM), aztreonam (AZT), piperacillin-tazobactam (PIP/TAZ), imipenem (IPM), meropenem (MEM), gentamicin (GM), tobramycin (TOB), fradiomycin (FRM), amikacin (AMK), ciprofloxacin (CIP), ofloxacin (OFLX), lomefloxacin (LFLX), levofloxacin (LVLX), orbifloxacin (OBFX), enrofloxacin (ERFX), and florfenicol (FF). The isolates were classified into three categories, namely susceptible, intermediate, and resistant, based on the breakpoints described by CSLI for Vet-08 and M-100-30th ED. The MIC of IPM, MEM, and COL were also determined against *P. aeruginosa* isolates using the broth microdilution technique, according to the relevant CLSI guidelines. Based on criteria provided by previous classification (39), the classification of the *P. aeruginosa* isolate as either multidrug-resistant (MDR), extensively drug-resistant (XDR), pan-drug resistant (PDR), or non-MDR (non-multidrug resistant) was based on its susceptibility to various antimicrobial agents. Specifically, an isolate was deemed MDR if it showed resistance to at least one agent in three or more antimicrobial categories. Conversely, an isolate was classified as XDR if it was susceptible to at least one agent in two or fewer categories. An isolate was labeled as PDR if it was resistant to all available antimicrobials. Finally, an isolate was considered non-MDR if it did not meet the criteria for MDR classification (39).

### Multilocus sequence typing analysis

The bacterial DNA was extracted using the Kaneka Easy DNA Extraction Kit (Version 2, Kaneka, Tokyo, Japan). The extracted DNA was purified using a Wizard Genomic DNA Purification kit (Promega, Tokyo, Japan). The sequences for the seven housekeeping genes *acsA*, *aroE*, *guaA*, *mutL*, *nuoD*, *ppsA*, and *trpE* of *P. aeruginosa* were amplified *via* PCR using appropriate primer sets (32). The PCR product was sequenced with a Big Dye Terminator 3.1 Cycle Sequencing Kit using an ABI 3100 genetic analyzer (Applied Biosystems).

The allelic number and ST were determined using the MLST database.^1^ If no matches were found in the database, STs were newly registered to the database (Supplementary Table S1). For phylogenetic analysis, the concatenated sequences of the seven housekeeping genes of all STs for *P. aeruginosa* (including the STs of the current study) were downloaded from the MLST website.^2^ The sequences were aligned using Multiple Sequence Comparison by Log-Expectation, and the phylogenetic tree was generated by the neighbor-joining method, using MEGA software version 10.2.4. The tree was visualized by the online iTOL software version 6.1.1.^3^

### Data analysis

All data in the current study were statistically analyzed using a computer software. For detection of age prevalence in the affected dogs, data were checked by the Mann–Whitney U test (GraphPad Software, Inc., San Diego, California). In all analyzes, *p* < 0.05 was considered statistically significant. To avoid any discrepancy in the results of the antimicrobial resistance profile of each isolate, all disk diffusion and MIC tests were performed in triplicate. The genotype structure of *P. aeruginosa* isolates was analyzed by the goeBURST algorithmic method using PHYLOViZ v2.0 software (40).^4^ The clonal relatedness among the STs of the 29 clinical isolates was checked based on their allelic number. STs were categorized into the same group if they had mutually identical alleles at six of the seven loci with one other member of the group. Conversely, the STs that did not share identical alleles in at least two out of seven loci were considered singleton STs. Using the iTOL software version 6.1.1 (see footnote 3), a heatmap with hierarchical clustering was created to show the overall distribution of antibiotic resistance phenotypes in the isolates.

## Results

### Description of dog cohort for *Pseudomonas aeruginosa* isolation in this study

Information regarding the affected dogs with otitis is described in Table 1. All 29 dogs included in the study were found to have 29 *P. aeruginosa* isolates (100%) in their ear canals. In this cohort, 65.5% (19/29) of dogs with otitis were male either intact (*n* = 6) or castrated (*n* = 13). No breed predisposition was observed for otitis. However, the incidence of the disease was significantly higher (Mann–Whitney U test, *p* < 0.0001) in senior dogs (above 7 years), who had reached the last quarter of their life expectancy as indicated in a previous study (41), with a mean ± standard deviation (SD) of 11.28 ± 2.37 than in mature adult ones with a mean (±SD) of 5.57 ± 1.39 years old. In addition, 37.9% (11/29) of the dogs with otitis had been previously treated with fluoroquinolones.

### Antimicrobial susceptibility profile

**Figure 1:**
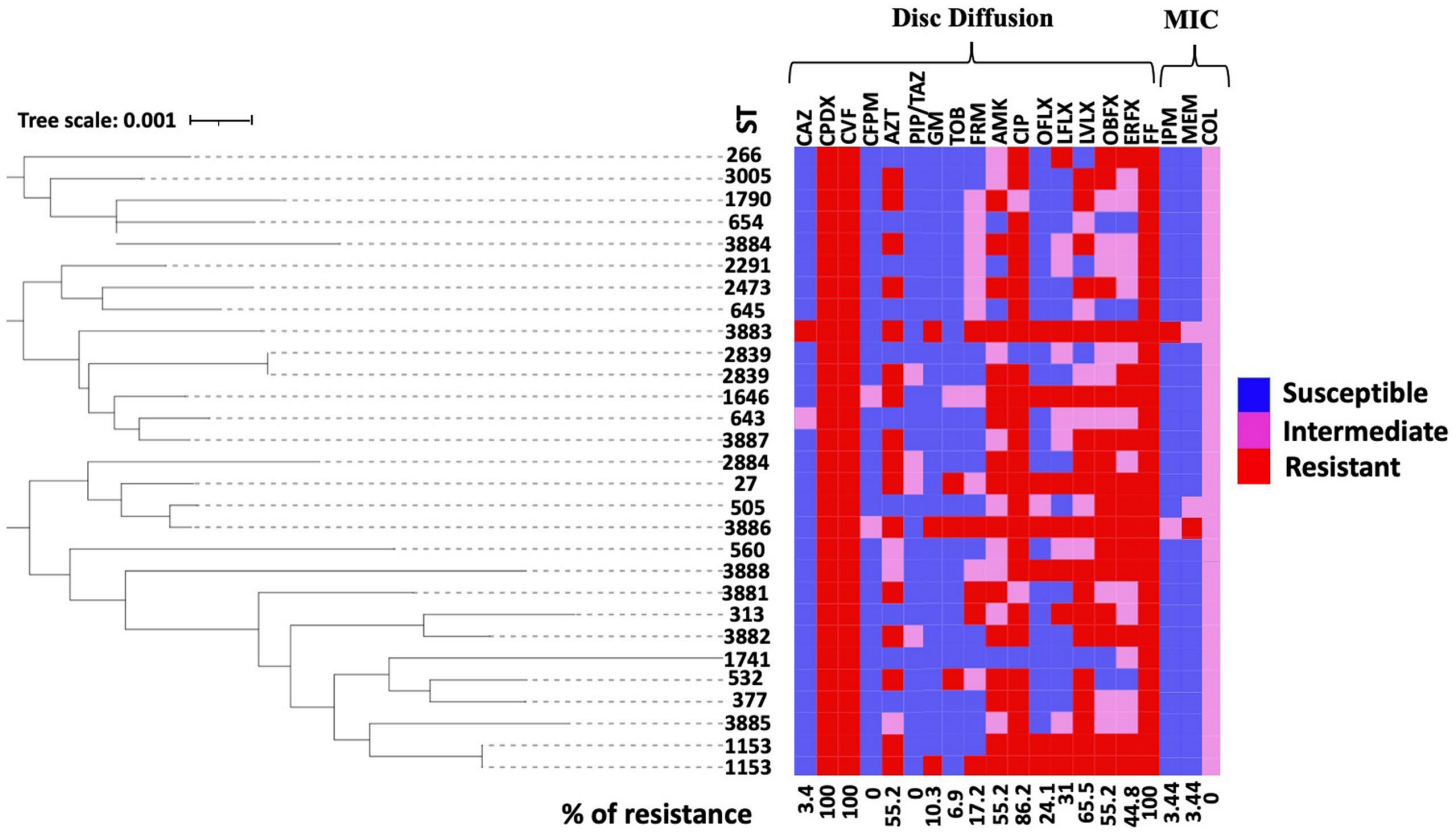
Antimicrobial susceptibility pattern of the 29 isolates isolated from the ear canals of dogs. A rectangular neighbor-joining tree was constructed based on the MLST data of all isolates. A heat map representing the antimicrobial susceptibility profile for each strain was annotated to the phylogenetic tree. Antimicrobial tests for colistin (COL), ceftazidime (CAZ), cefpodoxime (CPDX), cefovecin (CFV), cefepime (CFPM), aztreonam (AZT), piperacillin-tazobactam (PIP/TAZ), imipenem (IPM), meropenem (MEM), gentamicin (GM), tobramycin (TOB), fardiomycin (FRM), amikacin (AMK), ciprofloxacin (CIP), ofloxacin (OFLX), lomefloxacin (LFLX), levofloxacin (LVLX), orbifloxacin (OBFX), enrofloxacin (EREX), and florfenicol (FF) were performed using the disk diffusion method and MIC test based on CSLI. The figure shows the resistance status of antimicrobials (red, resistant; pink, intermediate; blue, sensitive).

The detailed number and percentage of frequency of susceptibility, intermediate susceptibility, and resistance toward each antimicrobial agent are expressed in Table 2. All the isolates: *n* = 29/29 (100%) expressed resistance phenotype toward CPDX, CFV, and FF. Additionally, the isolates of *P. aeruginosa* showed variable frequencies of resistance to the group of fluoroquinolones antibiotics CIP: *n* = 25/29 (86.2%), ERFX: *n* = 13/29 (44.8%), LVLX: *n* = 19/29 (65.5%), OFLX: *n* = 7/29 (24.1%), LFLX: *n* = 9/29 (31%), and OBFX: *n* = 16/29 (55.2%). In addition, *P. aeruginosa* isolates harbored resistance ranging from 10.3 to 55.1% toward the aminoglycoside group of antibiotics, such as GM: *n* = 3/29 (10.3%), TOB: *n* = 3/29 (10.3%), AMK: *n* = 16/29 (55.2%), and FRM: *n* = 5/29 (17.2%). In contrast, the isolates showed the lowest frequency of resistance toward cephalosporins CFPM: *n* = 0/29 (0.0%) and CAZ: *n* = 1/29 (3.4%) and PIP/TAZ: *n* = 0/29 (0.0%) (Figure 1). The frequency of resistance toward carbapenems antibiotics was 3.4% for IPM and MEM. All isolates showed intermediate susceptibility to COL (Table 3). Additionally, in terms of susceptibility to antimicrobials, 10/29 (34.5%) and 17/29 (58.6%) of *P. aeruginosa* isolates were classified as MDR and XDR, respectively, while 2/29 were non-MDR isolates. The antibiotic pattern and resistance profile for each isolate are shown in Table 1.

**Table 2:** Antimicrobial susceptibility percentage (susceptible, intermediate, and resistant) of the 29 *P. aeruginosa* isolates obtained from the ear canals of dogs.

Antimicrobial agent	Disk concentration (μg)	Breakpoints (mm; ≤S/I/R<)^a^	Number of susceptible isolates (%)	Number of Intermediate isolates (%)	Number of resistant isolates (%)
Ceftazidime (CAZ)	30	18/15–17/14	27 (93.1)	1 (3.4)	1 (3.4)
Cefpodoxime (CPDX)	10	21/18–20/17	0 (0.0)	0 (0.0)	29 (100)
Cefovecin (CFV)	10	21/18–20/17	0 (0.0)	0 (0.0)	29 (100)
Cefepime (CFPM)	30	18/15–17/14	27 (93.1)	2 (6.9)	0 (0.0)
Aztreonam (AZT)	30	22/16–21/15	10 (34.5)	3 (10.3)	16 (55.2)
Piperacillin-Tazobactam (PIP/TAZ)	110	21/15–20/14	25 (86.2)	4 (13.8)	0 (0.0)
Gentamicin (GM)	10	15/13–14/12	26 (89.7)	0 (0.0)	3 (10.3)
Tobramycin (TOB)	10	15/13–14/12	26 (89.7)	1 (3.4)	2 (6.9)
Fradiomycin (FRM)	10	15/13–14/12	14 (48.3)	10 (34.5)	5 (17.2)
Amikacin (AMK)	30	17/15–16/14	4 (13.8)	9 (31)	16 (55.2)
Ciprofloxacin (CIP)	5	21/16–20/15	2 (6.9)	2 (6.9)	25 (86.2)
Ofloxacin (OFLX)	5	16/13–15/12	21 (72.5)	1 (3.4)	7 (24.1)
Lomefloxacin (LFLX)	10	22/19–21/18	13 (44.8)	7 (24.1)	9 (31)
Levofloxacin (LVLX)	5	17/14–16/13	4 (13.9)	6 (20.6)	19 (65.5)
Orbifloxacin (OBFX)	10	23/17–22/16	4 (13.9)	9 (31)	16 (55.2)
Enrofloxacin (ERFX)	10	23/17–22/16	3 (10.3)	13 (44.8)	13 (44.8)
Florfenicol (FF)	30	16/11–15/10	0 (0.0)	0 (0.0)	29 (100)

^a^S, susceptible; I, intermediate; R, resistant.

**Table 3:** Minimum inhibitory concentration (MIC) for imipenem, meropenem, and colistin for the 29 *P. aeruginosa* isolates obtained from the ear canals of dogs.

Antimicrobial agent	MIC breakpoints (μg/mL)			Results of MIC		
	*S*	*I*	*R*	Susceptible strains (%)	Intermediate strains (%)	Resistant strains (%)
Imipenem IPM	≤2	4	≥8	27 (93.1)	1 (3.4)	1 (3.4)
Meropenem MEM	≤2	4	≥8	26 (89.7)	2 (6.9)	1 (3.4)
Colistin COL	-	≤2	≥4	0 (0.0)	29 (100)	0 (0.0)

### Clonal complexes distribution

**Figure 2:**
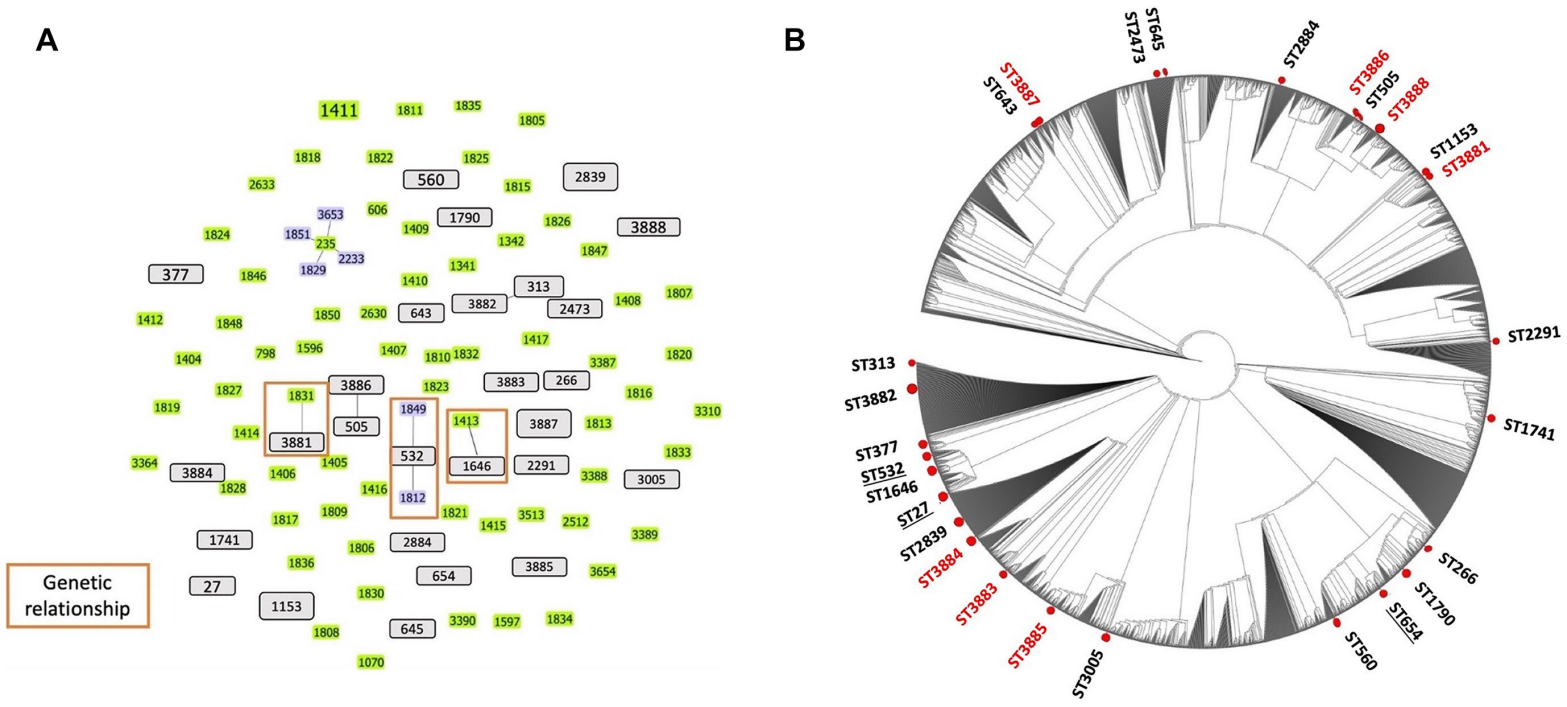
Population structure of *Pseudomonas aeruginosa* strains: **(A)** Genetic relatedness between all known sequence types (STs) and the canine *P. aeruginosa* STs identified in Japan. Three clonal complexes were displayed *via* single locus variant analysis using goeBURST software (orange rectangular). Gray bars represent all STs identified in this study from dogs. Green and blue (founder ST) bars indicate the STs identified in Japan based on the pubMLST database. **(B)** The phylogenetic tree represents the distribution of the STs identified in this study from dogs among all known STs from the pubMLST database. Red circles indicate the positions of STs in this study (red STs are new STs identified). No cluster formation was observed among the STs of *P. aeruginosa* isolated from dogs. Underlined STs represent the high-risk clones of *P. aeruginosa* identified from dog isolates in this study.

Using the MLST analysis, 27 STs were identified among the 29 isolates from the ear canals of dogs affected with otitis. Two STs were assigned to four isolates: ST 1153 was assigned to PA 24667 and PA 30323 isolates, while ST2839 was assigned to PA 27976 and PA 33599 isolates. The same STs mean identical sequences in all housekeeping genes. Other STs were assigned for other isolates. We then analyzed the CCs using these STs. The present STs did not exhibit a CC formation among the isolated strains except for two groups clonally related by a single locus variant: ST313 with ST3882 and ST505 with ST3886. The remaining 25 STs were classified as singletons (Figure 2A), indicating the high genetic diversity between the isolates. The genetic relatedness of the strains isolated in this study with the STs prevalent in Japan was examined. A relationship was observed between two STs, ST3881 and ST1646, which are clonally related by a single locus variant to the previously identified ST1831 and ST1413 from Japan, respectively, while ST532 is the group founder of two Japanese STs, ST1812 and ST1849 (Figure 2A). Also, three previously identified human high-risk clones were identified in the current study: ST27, ST532, and ST654.

The present study conducted a phylogenetic analysis using concatenated sequences of MLST alleles, which involved both the STs of this study and the entire MLST database as of the date of analysis. The circular tree obtained from this analysis indicates that the STs examined in our study display diversity and are dispersed among all known STs for the *P. aeruginosa* organism, without forming any distinct clusters. The results of this analysis are illustrated in Figure 2B. Furthermore, the heat map for antimicrobial susceptibility patterns was annotated to the rectangular phylogenetic tree of the STs to examine the dissemination of resistant phenotypes on the tree. The antibiotic resistance phenotypes (i.e., susceptible, intermediate, and resistant) were distributed in all clusters. The highly resistant strains did not vary meaningfully among clusters (Figure 1).

## Discussion

It has been reported in various countries that opportunistic infection causing agents, such as *P. aeruginosa* and other companion-animal-borne organisms, are developing antibiotic resistance (42, 43). The spread of bacteria resistant to antibiotics from animals to people is a major public health concern, especially when those bacteria are resistant to antimicrobials used in human medicine (23). The One Health approach emphasizes the necessity of *P. aeruginosa* antimicrobial resistance surveillance, particularly in human-animal-environment interactions. However, studies on continuous monitoring of antimicrobial patterns and the genetic characterization of *P. aeruginosa* isolates from dogs are limited in Japan. From the review of the frequency of resistance to the 20 antibiotics demonstrated in our study, we concluded that the therapeutic lines for these isolates are challenging. The high variation in resistance frequency toward commonly used veterinary antibiotics such as fluoroquinolones (ranging from 24.1 to 86.2%), makes choosing a suitable antimicrobial difficult, and this could be attributed to the discrepancy between different methods used for checking susceptibility (the disk diffusion test in our study). Nonetheless, when otitis associated with a *P. aeruginosa* infection is diagnosed, an antibiotic sensitivity test remains the reliable method for identifying and guiding the optimal treatment (44). Compared to the scientific investigations on *P. aeruginosa* isolated from dogs, the antibiotic resistance profile was slightly higher in the isolates of the present study, with the appearance of a high frequency of MDR and XDR isolates (36, 37). This increase in the number of isolated resistant strains in this study may be attributed to the well-recognized resistant and sometimes multi-resistant nature of the bacterium against the antibiotics commonly used in the veterinary field. Hence, the choice of antimicrobials to treat the disease in clinical settings is limited (45). The overproduction of chromosomally encoded AmpC genes, the outer membrane porin OprD, and the multidrug efflux system are the predominant therapeutic challenges of this organism (46). The phylogenetic tree showed that the antimicrobial resistance phenotypes are distributed among all strains without a significant cluster formation, indicating a weak resistance-related genetic association between the current isolates. Notably, two strains possess the same STs (ST2839 and ST1153) and are shown to have variable resistant rates to antibiotics. However, they possess a common susceptibility to CAZ, CFPM, GM, and TOB, suggesting the possible treatment of strains sharing identical genetic material.

Interestingly, the Infectious Diseases Control Law in Japan did not describe a carbapenem-resistant isolate of *P. aeruginosa* and MDR isolates originating from companion animals. However, the current study detected that 34.5 and 58.6% of *P. aeruginosa* isolates were classified as MDR and XDR, emphasizing the need for regular resistance investigation. The rates of GM and CAZ resistance in our study was 10.3, and 3.4%, respectively, and are similar to that reported previously against *P. aeruginosa* in dogs, which is 19.4 and 1.4%, respectively (37, 47, 48). The CAZ, GM, PIP/TAZ, CFPM, and TOB retained bactericidal activity against the isolates from the ear canals of dogs.

Companion animals represent an insufficiently researched reservoir of *P. aeruginosa* strains that exhibit antimicrobial resistance. In instances of MDR-*P. aeruginosa* infections in humans, polymyxin (colistin) and carbapenems (imipenem and meropenem) are recognized as the ultimate therapeutic options (49). In this study, we investigated the prevalence of resistant strains of *P. aeruginosa*, isolated from dogs in Japan, toward COL, IPM, and MEM using the MIC test, which is reliable and accurate for these antimicrobials. Our investigation revealed that all *P. aeruginosa* isolates exhibited intermediate susceptibility to colistin (COL). This finding emphasizes the need for continuous monitoring of isolated strains from companion animals in Japan. At the same time, the resistance toward carbapenems is also crucial. The carbapenems resistant isolates of *P. aeruginosa* have been detected by the MIC test and found to be 3.4% for IPM and MEM. The epidemiological investigation on the increase in the frequency of carbapenems resistance was seen to vary from 0.0% in 2003–2010 (37), 0.5% in 2014–2015 (36), 6.67% in 2017–2018 (50) to 3.4% in 2020 (the current study). In Japan, the carbapenem-resistant *P. aeruginosa* isolates from companion animals were found to fluctuate over time (50), indicating that a continuous survey of carbapenem-resistant *P. aeruginosa* from the pets’ environment is necessary. Although the carbapenems resistance status in Japan is not a crisis as reported by the Japanese Veterinary Antimicrobial Resistance Monitoring (JVARM) system, recent scientific studies (36, 37, 50) reported an increase in the carbapenem-resistant isolates from dogs and cats. However, the use of antimicrobials in Japan seems to be prudent, and this increase might be due to the prescriptions of such medicines by local drug suppliers, personnel importation of drugs, and the use of human antimicrobials in the treatment of companion animals, which are not monitored under the JVARM and are excluded from calculations (51). These findings indicate that the veterinary niches, especially the dog-human interface, represent a potential reservoir of resistant *P. aeruginosa* for animals and humans. The use of COL in human medicine was relatively low owing to its neurotoxicity and nephrotoxicity (52). However, in veterinary and human clinical practice, COL has been reintroduced to treat pathogens resistant to other available antimicrobials. The recommended treatment of otitis in dogs includes recognizing and managing the predisposing factors, ear cleaning, topical treatment, and systemic injection of antimicrobials (if necessary). Polymyxins, including COL, has been introduced in some countries as authorized active ingredients for topical ear application in canine otitis treatment (43) and for intravenous treatment of food-producing animals and equine septicemia (53, 54). Restriction on the use of COL, IPM, and MEM in the veterinary section is required for saving these antibiotics as the last lines of defense against MDR-*P. aeruginosa* in humans, especially cystic fibrosis (49, 55).

The inappropriate use of antibiotics may induce an annual increase in antibiotic resistance, particularly for antibiotics considered crucial therapeutic agents for treating *P. aeruginosa* infections in humans. The high level of resistance toward fluoroquinolones (i.e., CIP and ERFX) among isolates that originated from dogs is alarming. The ERFX antibiotic is a potential therapeutic option for the treatment of many human diseases (56). The Nippon AMR One Health Report^5^ stated in 2021 that fluoroquinolones such as ERFX and OBFX have been used in a persistent amount (between 0.81 and 0.91 ton every year from 2013 to 2018) for treatment of dogs and cats. The amount of CIP used, which is an active metabolite of ERFX, cannot be measured because its prescription has been restricted for veterinarians in Japan. Thus, attention should be paid to monitoring the jumping of resistance to ERFX and CIP. Collectively, the isolated *P. aeruginosa* strains from dogs in the current study exhibited a high frequency of antibiotic resistance, yet CFPM, GM, TOB, PIP/TAZ, and CAZ possess the ability to kill the *P. aeruginosa* isolated from the ear canal of affected dogs. A widely utilized approach to preserve susceptibility to these therapeutic agents for clinical purposes is combining phage therapy with alternative antimicrobial methods, such as phage-antibiotic synergy. This method is particularly effective against drug-resistant bacteria (57).

Notably, animals, particularly companion animals have been recognized as potential reservoirs of multidrug-resistant bacteria and resistance genes that can be transmitted to humans (58–60). The genetic analysis of *P. aeruginosa* isolates from dogs provides important information about the geographical distribution of these isolates in the veterinary community related to humans (59). In the present study, three previously identified human high-risk clones (ST532, ST27, and ST654) were identified from the isolates of infected dogs in this study, which represent their ability to colonize in different niches including the animal environment, and is indicative of a possible zoonotic transmission to humans (61). We chose the MLST technique for our study to easily distinguish the strains of *P. aeruginosa* and compare STs recovered from dogs with that circulating in the same geographical area, i.e., Japan. Our study supports the nonclonal epidemic structure of *P. aeruginosa* strains (62–64), whereas the genetic relatedness between some clones isolated from dogs (i.e., ST3881, ST1646, and ST532) and the Japanese STs isolated from wounds and urinary tract infections in humans (i.e., ST1831, ST1413, ST1812, and ST1849) support the theory of horizontal transmission of *P. aeruginosa* from dog to human. Previous studies have reported MDR-*P. aeruginosa* isolates from an infected dog, its owner, and the domestic environment, which suggests the occurrence of zoo anthroponotic transmission (65, 66). In addition, the phylogenetic analysis of the resulting data facilitates a clear demonstration of the relative intraspecies evolutionary position of dogs-inhabiting strains indicated by the non-clustered distribution of dog STs among the whole MLST database of the *P. aeruginosa*. The findings of the current study (i.e., the genetic association between dog STs and those circulating in humans in Japan and the dog STs that matched the STs in the MLST database isolated from different clinical samples) promoted the concept that *P. aeruginosa* isolates are merely environmental strains that transmit to humans after becoming environmentally adapted. Here, we can support the hypothesis of the zoonotic transmission from either the dog itself or the environment of an infected dog to his immunocompromised owner, especially in the presence of pet-human life sharing. The current findings hypothetically support the previous reports of *P. aeruginosa* transmission, either directly or indirectly through contamination of environmental objects (67–69). Since pet ownership, especially dogs and cats, become progressively popular in Japan, awareness and knowledge about related public health concerns should be raised. While we have made efforts to ensure the reliability of each experimental result presented in this study, it is important to acknowledge certain limitations that may affect the interpretation of the empirical findings. These limitations include the small sample size, which needs to be addressed in future studies to further support and strengthen the conclusions drawn.

## Conclusion

Continuously updating the antimicrobial resistance profiles is necessary to establish the appropriate antibiotic drugs for treating *P. aeruginosa* infections in dogs and monitoring the emergence of new clones of *P. aeruginosa* from the pets’ environment. In addition, our study showed that CFPM, PIP/TAZ, CAZ, TOB, and GM could be reliable therapeutic options for treating pseudomonal canine otitis.

## Data availability statement

The original contributions presented in the study are included in the article/Supplementary materials, further inquiries can be directed to the corresponding author.

## Author contributions

AE, JU, and KN conceived and designed the study, and substantially revised the paper. AE, JU, KG, KIY, and YT carried out the experiments, data analysis, and acquisition. AE, II, RR, WN, TT, and KK drafted the original manuscript. JU, KID and KN supervised the study. All authors read and approved the final manuscript.

## Funding

AE has received financial support from Damanhour University and the Mission Sector, Ministry of Higher Education, Egypt to accomplish the current study (program 2018/2019).

## Conflict of interest

TT was employed by Zenoaq Co. Ltd.

The remaining authors declare that the research was conducted in the absence of any commercial or financial relationships that could be construed as a potential conflict of interest.

## Publisher’s note

All claims expressed in this article are solely those of the authors and do not necessarily represent those of their affiliated organizations, or those of the publisher, the editors and the reviewers. Any product that may be evaluated in this article, or claim that may be made by its manufacturer, is not guaranteed or endorsed by the publisher.

## Footnotes

^1^https://pubmlst.org/bigsdb?db=pubmlst_paeruginosa_seqdef

^2^https://pubmlst.org/paeruginosa/; accessed on 5 February 2022

^3^https://itol.embl.de/

^4^http://goeBURST.phyloviz.net

^5^https://www.mhlw.go.jp/content/10900000/000885373.pdf
