# Supplementary material for: Antimicrobial resistance and genotyping of Pseudomonas aeruginosa isolated from the ear canals of dogs in Japan
Source: Front Vet Sci. 2023 Jul 20;10:1074127. doi: 10.3389/fvets.2023.1074127 (PMC10397403; doi:10.3389/fvets.2023.1074127)
Supplement: Supplementary file 1 [file Table_1.DOCX]

Supplementary Material

**Supplementary Table 1** List of alleles and sequence types (STs) of the 29 isolates of *Pseudomonas* *aeruginosa* obtained from the ear canals of dogs.

| **Strain ID** | ***acs*A** | ***aro*E** | ***gua*A** | ***mut*L** | ***nuo*D** | ***pps*A** | ***trp*E** | **STs** |
| --- | --- | --- | --- | --- | --- | --- | --- | --- |
| PA 22861 | 2 | 4 | 5 | 3 | 8 | 6 | 7 | 3881 |
| PA 23303 | 47 | 8 | 7 | 6 | 8 | 11 | 7 | 3882 |
| PA 23405 | 39 | 5 | 1 | 93 | 4 | 46 | 7 | 3883 |
| PA 23824 | 28 | 5 | 12 | 3 | 4 | 1 | 18 | 643 |
| PA 23943 | 17 | 5 | 26 | 3 | 4 | 4 | 26 | 654 |
| PA 24223 | 28 | 89 | 11 | 43 | 103 | 92 | 3 | 3884 |
| PA 24374 | 25 | 20 | 7 | 3 | 3 | 3 | 7 | 2291 |
| PA 24608 | 6 | 5 | 5 | 3 | 3 | 13 | 1 | 645 |
| PA 24667 | 2 | 4 | 5 | 31 | 1 | 6 | 11 | 1153 |
| PA 25065 | 5 | 1 | 109 | 3 | 12 | 1 | 47 | 1741 |
| PA 25268 | 1 | 89 | 80 | 3 | 4 | 10 | 3 | 1790 |
| PA 27532 | 15 | 5 | 7 | 3 | 2 | 7 | 68 | 1646 |
| PA 27567 | 83 | 3 | 6 | 13 | 2 | 6 | 26 | 3885 |
| PA 27976 | 7 | 89 | 94 | 54 | 14 | 87 | 7 | 2839 |
| PA 30241 | 103 | 8 | 5 | 5 | 1 | 6 | 4 | 377 |
| PA 30323 | 2 | 4 | 5 | 31 | 1 | 6 | 11 | 1153 |
| PA 30580 | 47 | 8 | 7 | 6 | 8 | 11 | 40 | 313 |
| PA 32450 | 36 | 5 | 36 | 61 | 4 | 4 | 52 | 3005 |
| PA 33146 | 6 | 5 | 11 | 131 | 3 | 53 | 1 | 2473 |
| PA 33343 | 188 | 5 | 11 | 205 | 44 | 7 | 7 | 2884 |
| PA 33427 | 16 | 5 | 11 | 72 | 44 | 7 | 52 | 266 |
| PA 33543 | 5 | 5 | 57 | 13 | 1 | 40 | 3 | 560 |
| PA 33568 | 6 | 20 | 1 | 11 | 4 | 4 | 2 | 505 |
| PA 33599 | 7 | 89 | 94 | 54 | 14 | 87 | 7 | 2839 |
| PA 33670 | 6 | 5 | 6 | 7 | 4 | 6 | 7 | 27 |
| PA 34383 | 6 | 89 | 1 | 11 | 4 | 4 | 2 | 3886 |
| PA 34599 | 5 | 4 | 5 | 5 | 5 | 20 | 4 | 532 |
| PA 35113 | 7 | 5 | 7 | 3 | 5 | 1 | 7 | 3887 |
| PA 35671 | 119 | 10 | 3 | 5 | 8 | 6 | 77 | 3888 |

STs highlighted in grey indicate that they are first described in the present study. Underlined STs indicate that they represent two isolates.
